# Practical Evaluation of Printed Strain Sensors Based on Long-Term Static Strain Measurements

**DOI:** 10.3390/s21144812

**Published:** 2021-07-14

**Authors:** Daniel Zymelka, Kazuyoshi Togashi, Toshihiro Takeshita, Takahiro Yamashita, Takeshi Kobayashi

**Affiliations:** 1 National Institute of Advanced Industrial Science and Technology, Tsukuba 305-8564, Japan; toshihiro-takeshita@aist.go.jp (T.T.); takahiro-yamashita@aist.go.jp (T.Y.); takeshi-kobayashi@aist.go.jp (T.K.); 2 NMEMS Technology Research Organization, Tokyo 101-0026, Japan; ktogashi@nmems.or.jp

**Keywords:** flexible printed electronics, strain sensors, static strain analysis, large area electronics

## Abstract

Recent progress in printable electronics has enabled the fabrication of printed strain sensors for diverse applications. These include the monitoring of civil infrastructure, the gradual aging of which raises concerns about its effective maintenance and safety. Therefore, there is a need for automated sensing systems that provide information on the performance and behavior of engineering structures that are subjected to dynamic and static loads. The application of printed strain sensors in structural health monitoring is of growing interest owing to its large-area and cost-effective fabrication process. Previous studies have proven the suitability of printable strain sensors for dynamic strain measurements on bridges; however, the analysis of the long-term stability of printed sensors during static strain measurements is still lacking. Thus, this study aims to assess the long-term stability of printed strain sensor arrays and their suitability for the static strain analysis of large civil structures. The developed sensors and a dedicated wireless data acquisition system were deployed inside a gravity dam, which was selected as the field test environment. This test environment was chosen owing to the relatively stable temperature inside the dam and the very slow static strain changes associated with periodic water level changes. The results exhibited an average signal drift of 20 μϵ over 127 days. One of the sensor arrays was installed on a small crack in the dam structure; it showed that the sensors can track static strain changes owing to variations in the crack opening, which are related to the water level changes in the dam. Overall, the results of the developed sensors exhibit good strain sensitivity and low signal drift. This indicates the potential suitability of printed sensors for applications in the static strain analysis of engineering structures.

## 1. Introduction

The development of sensing systems for the diagnosis and health assessment of civil engineering structures is essential to ensure the safety of bridges, tunnels, buildings, pipelines, and dams. Such diagnosis and health assessment processes that utilize automated sensing systems that comprise networked sensors, data acquisition devices (DAQ), and diagnostic algorithms are usually described as structural health monitoring (SHM). More specifically, SHM refers to the process of implementing a damage detection strategy and systems to identify structural damage in analyzed engineering structures [1,2]. It involves the observation of a structure over time, using periodically sampled responses from a sensor (or array of sensors), and the evaluation of the current and future performance of the monitored structure [3].

Diverse sensing systems have been developed to monitor civil infrastructure. Such systems are typically based on the use of optical fibers [4,5,6], acoustic emission sensors [7], accelerometers [8,9], and conventional metal foil strain gauges [10,11]. The appropriate selection and configuration of a sensing system that incorporates one or multiple sensor types generally depends on the use case; that is, the type of monitored structure, the size of the investigated construction elements, and the materials used to build the analyzed object. Among all sensor types, the use of optical fibers has become especially popular due to its numerous advantages, such as the potential to multiplex many sensing points along a single optical fiber. Such sensors can be deployed over large distances, thereby providing information about structural integrity and local deformations (strain) along the entire length of a bridge or other large engineering structures. However, the sensing capability of fiber optic sensors is limited to the spatial size of the fiber, which must be considered when selecting a sensing system for a specific use case. Moreover, the low spatial resolution of optical fibers may be considered a drawback for two-dimensional (2D) strain analysis intended for crack detection in the proximity of sensitive construction elements in investigated structures. This has already been identified in a previous study [12], wherein the potential advantages of direct sensing systems were demonstrated over indirect systems. Various concepts for distributed sensor arrays have been previously investigated for applications in 2D strain analysis. For example, an array of commercial metal foil strain gauges bonded to a flexible substrate detected and quantified cracks in structural materials [13]. Further studies presented another type of sensing sheet that incorporated constantan-based (Cu-Ni alloy) strain sensors that were fabricated on a flexible substrate using a photolithography etching process [14]. The signal stability of the sensing sheet was analyzed in a laboratory test, and the results revealed a 3 μϵ drift over two days of analysis. Moreover, the sensing sheet and dedicated data acquisition system were deployed on a concrete bridge for a several-hour-long field test. Other studies on the development of large-area sensor arrays have demonstrated capacitive sensors [15,16] and sensor arrays that utilize lead zirconate titanate (PZT) piezoelectric sensors [17,18,19].

Another group of sensors that is worth considering consists of printed strain sensors. Their principle of operation is similar to that of commercially available strain gauges. When a sensor is subjected to mechanical deformation, the electrical resistance of the material used to fabricate the sensor changes as a function of the applied strain. However, the primary difference compared with standard strain gauges is the fabrication process. Recent progress in additive manufacturing, which is widely used for flexible printed electronics [20], enables new possibilities for large-area and cost-effective fabrication, which is highly desirable for applications in SHM. The available printing methods and materials allow the fabrication of sensors as well as passive components with their entire wiring systems on various types of lightweight flexible substrates [21]. Owing to the benefits of printed electronics, printed strain sensors have been studied for various applications [22,23,24,25,26], including SHM [27,28,29,30].

Although there are several published reports on printed strain sensors for SHM, there is still a lack of experimental data recorded under practical conditions. However, such data are necessary to assess the long-term durability and performance of printed strain sensors for applications in real-life scenarios of SHM in various engineering structures. We addressed this gap in our previous studies by deploying printed strain sensors on highway bridges and analyzing their performance. In the first field test, an array of 16 printed strain sensors was bonded to a bridge structure to assess its long-term durability [31]. At that time, the sensors were not equipped with any data acquisition system deployed on the bridge. The measurements were performed periodically, every few months, using a portable DAQ. The results showed that the developed printed sensors could withstand at least one year of continuous exposure to typical traffic conditions on a highway bridge. Although the results of the first field test were very promising, the sensor array was redesigned for the next test and equipped with a wireless data acquisition system that was connected to the Internet [32]. The modifications added the feature of remote control over the entire sensing system. The number of sensors in the array was increased to 25, and the sensors and substrates were kept at the same size. Thus, the density of the sensor pattern increased, thereby providing more sensing points. These experiments were intended to collect dynamic strain data on a highway bridge during a five-month-long continuous experiment. Because the dynamic strain measurements generated a large volume of data, the measurements were intermittent; that is, dynamic strain measurements of 2.5 min were automatically performed every 4 h within a period of 5 months. Based on the collected measurements, the durability of the sensors and the traffic conditions were analyzed at various periods of the day and on weekdays. Further work demonstrated that the layout of the sensors in the array can be easily redesigned to monitor the local strain distribution around crack-stop holes, which are used in bridge construction to hinder crack propagation [33].

Previous studies have demonstrated the good performance of printed strain sensors. Nonetheless, the analysis primarily focused on the dynamic response of the printed sensors to traffic on highway bridges. However, within the framework of SHM, static strain measurements are equally important as dynamic strain analysis, especially when the sensors are intended for the analysis of large engineering structures other than bridges. Thus, in this study, printed strain sensor arrays and a wireless data acquisition system were deployed inside a gravity dam. The dam was chosen for the field test owing to the relatively stable temperature inside the dam and the very slow static strain changes associated with periodic water level changes in the dam. Thus, the long-term drift of sensors could be analyzed without the influence of sudden environmental fluctuations, and the sensors were subjected to very slow, gradual static strain changes. A specially prepared network of wireless signal repeaters was used for remote measurements, and they were automatically controlled from outside the dam. An assessment of the long-term stability of printed sensors and their suitability for practical implementation in static strain analysis was conducted for a period of four months.

## 2. Materials and Methods

The construction of the sensor array used in this study was similar to that used in our previous study, which focused on a dynamic strain analysis conducted on a highway bridge [32]. However, this section presents the most relevant information concerning the construction of the sensing system and newly implemented improvements. The main changes, compared with the previous study, were implemented in the construction of the DAQ, which was equipped with a set of wireless signal repeaters in this study. The use of signal repeaters was necessary to enable data transmission from the sensors installed inside the dam to a wireless receiver installed outside the dam. Moreover, the configurations of the data acquisition software and data processing were adapted to the analysis of static strains, which was the main purpose of this study.

### 2.1. Preparation of Sensors

**Figure 1:**
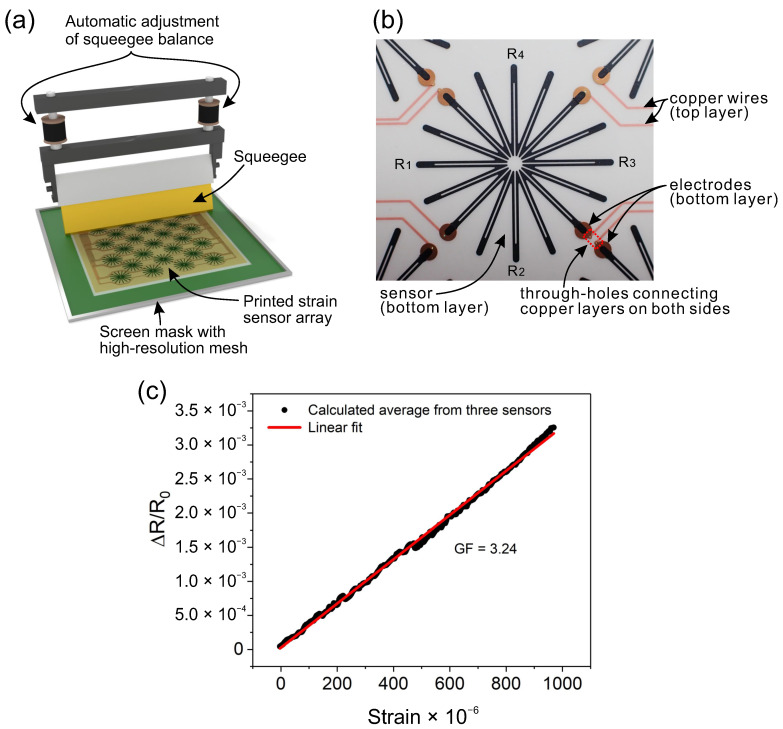
(**a**) Screen printing of the strain sensor array. (**b**) Back view of a single strain sensor incorporated into the array of 25 sensors. R${}_{1-4}$ show the particular resistive elements of the printed structure that complete the full-Wheatstone-bridge circuit. (**c**) Strain sensitivity calculated based on the average of three printed strain sensors.

Twenty-five strain sensors were screen-printed on the back of a polyethylene naphthalate (PEN) sheet with a double-sided copper wiring system (Figure 1a). The Cu-PEN-Cu laminate was prepared via the dry lamination of 50 μm-thick PEN between two 9 μm-thick copper sheets. The wire and electrode patterns were formed on both sides of the prepared laminate through the wet etching of Cu in ferric chloride. The interconnections between both sides of the Cu layers were formed by electroplating the through holes. A screen mask with a stainless steel mesh (HS-D 650/14, Asada Mesh, Osaka, Japan) was used for the high-resolution printing of the sensor pattern. The screen printer (Cube, Mino Group, Gifu, Japan) was equipped with a system to automatically adjust the squeegee balance, which is especially important in the fabrication process of large-area electronics. The sensors were made of carbon-based ink (DY-200L-2, Toyobo, Osaka, Japan), which provides good mechanical properties and strain sensitivity [32]. The external dimensions of the area covered by all the sensors were 10 × 10 cm. The diameter of a single sensor in the array was 16 mm (Figure 1b). After printing, the sensor array was cured in a conventional oven at 130 °C for 30 min. The sensor pattern and layout of the electrodes were designed such that each of the sensors represented the full-Wheatstone-bridge structure, which was necessary to compensate for the effect of expected temperature changes. It must be noted that sensors made of carbon-based materials are generally sensitive to temperature changes [28,34,35]. The advantages of the full-Wheatstone-bridge structure implemented in printed carbon-based strain sensors have been described in detail in our previous studies [31,32]. In contrast with conventional full-Wheatstone-bridge strain gauges that are composed of four individual sensors, our sensor has only one symmetrical structure that can complete the full-bridge circuit. This is especially important in terms of the uniformity of printing, which can be affected when printed patterns have some components that are oriented differently with respect to the printing direction [33]. During the measurements, the sensor array was bonded to the monitored structures using a 200 μm-thick epoxy-based adhesive sheet. The adhesive was custom-made within the framework of this study. The activation of the curing process of the adhesive requires the exposure of about 1 J/cm${}^{2}$ to ultraviolet light. The same type of adhesive sheet was used as a protection layer to cover the sensor array on the top side after it was attached to the dam. Thus, the sensors were fully sealed from both sides by the adhesive sheet, which protected them from humidity and moisture.

In addition to the 25 printed strain sensors in the array, an integrated temperature chip (TMP36, Analog Devices, Norwood, MA, USA) was installed on top of the copper laminate. The temperature sensor provided a local temperature analysis, which was recorded with the strain measurements. The output signals from all sensors were connected to the DAQ via flexible flat cables and connectors (502598-3393, Molex, Lisle, IL, USA).

### 2.2. Characterization of Sensors

Prior to the deployment of the sensing system inside the dam, the printed strain sensors were analyzed with a laboratory test to assess their strain sensitivity and the linearity of the output signal. To calculate the strain sensitivity, a printed strain sensor array was installed on a metal plate with three conventional sensors (KFG-10-120-C1-11L1M2R, Kyowa Electronic Instruments, Tokyo, Japan), which were used to obtain a reference strain measurement. The printed sensors were attached to the top of the plate, and the three conventional strain sensors were attached to the bottom at locations that corresponded to the positions of three printed sensors selected from the array. The plate was made of steel, and it had a length, width, and thickness of 70 cm, 12 cm, and 2 mm, respectively. One side of the plate was bolted to a stable support, and the other was connected to a hook that was bonded to a tensile test machine (FTN1-13A, Aikoh Engineering, Osaka, Japan). During the analysis, the plate with the attached sensors was subjected to bending deformation. Both types of sensors were subjected to equal strains during bending: positive on the top (printed sensors) and negative on the bottom (conventional sensors). The output signals from both types of sensors (voltage) were simultaneously recorded and analyzed using a specially prepared computer program (LabView, NI, Austin, USA). Based on the recorded data, the relative change in resistance ($\Delta R/R_{0}$) was calculated and compared with the corresponding strain (ε), which was measured using conventional strain gauges with a known sensitivity (gauge factor (GF)) of 2.09. The GFs of the printed sensors were calculated using the following equation:

(1) $$GF=\frac{\Delta R/R_{0}}{\varepsilon },$$

The collected data for the strain sensitivity analysis are shown in Figure 1c. The plot shows the average change in resistance, which was calculated from the three printed strain sensors during a full strain cycle up to approximately 1000 μϵ. The results show that the output signal was linear within the investigated strain range. The calculated GF was 3.24, which was sufficiently high for the practical use of the sensors. In addition to the good strain sensitivity and linearity, every strain sensor should exhibit a low sensitivity to temperature changes. The effectiveness of the developed printed full-Wheatstone-bridge sensors at compensating temperature changes was confirmed and demonstrated in our previous studies [32]; thus, such an analysis was not repeated for the purpose of this study.

### 2.3. Experimental Setup on the Dam

**Figure 2:**
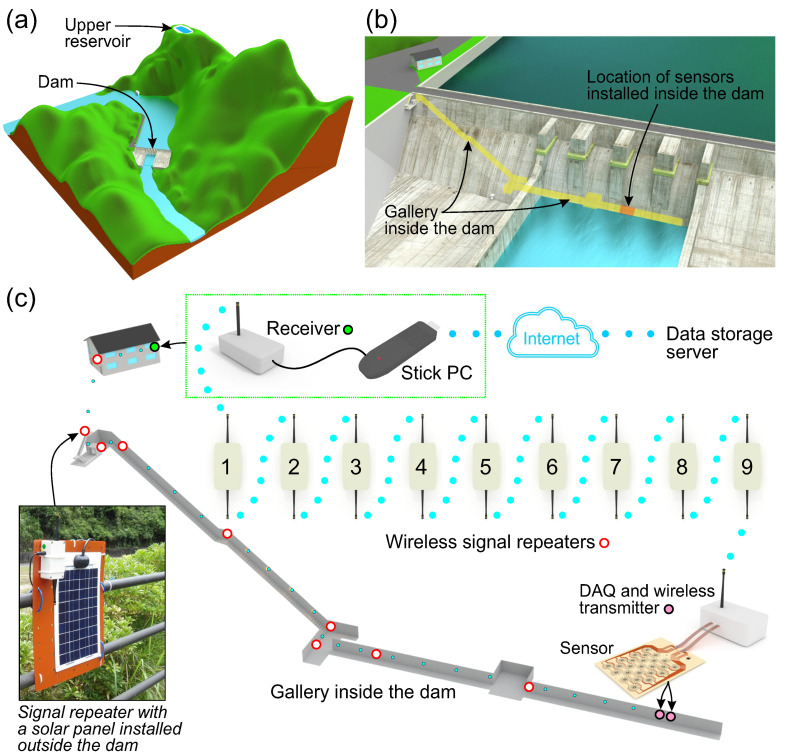
(**a**) Illustration of a pumped storage power station that consists of the dam and upper reservoir. Its operation regulates water level changes on the dam. (**b**) Location of sensors installed on the gallery wall inside the dam. (**c**) Sensing system that incorporates wireless signal repeaters installed along the gallery.

The sensors were installed inside the dam, which forms part of a pumped storage power station (Figure 2a). More specifically, two sensor arrays were bonded to the wall of a gallery located inside the dam (Figure 2b) and equipped with a dedicated wireless measuring system (Figure 2c). One of the sensors was attached to an undamaged surface to perform a long-term output signal stability test. The second sensor array was bonded to a surface on which a crack was found by engineers of the dam operator. The crack did not compromise the safety of the dam, and in this study, it was used only to verify if the developed sensors were sensitive enough to detect any strain changes caused by normal dam operation; that is, periodic water level changes that may cause variations in the crack opening. Thus, the printed strain sensors’ capability to detect damage based on static strain measurements was evaluated simultaneously with the stability test.

### 2.4. Data Acquisition System

Each sensor array was equipped with a wireless transmitter that incorporated a 25-channel, 24 bit analog-to-digital converter (ADC). The operation of the transmitters was controlled by specially designed computer software (LabView, National Instruments) that provided an interface with a wireless receiver, which was capable of sending commands to and receiving data from the transmitters connected to the sensors. To provide remote control of the entire sensing system, the computer was connected to a portable long-term evolution router. Therefore, the system could be controlled from any location that had access to the Internet. In this study, the sensors were installed inside a gravity dam, which is a large concrete engineering structure. Thus, the challenging part of this project was remote communication between the transmitters installed inside the dam and the receiver installed outside in a storage building located near the dam (Figure 2c). To enable communication, a system of networked signal repeaters was deployed along the gallery. In total, nine signal repeaters were used. Seven of these were installed inside the dam, and the other two were installed outside. The developed wireless data acquisition system was operated at a frequency of 920 MHz. Inside the dam, the power supply to the transmitters and signal repeaters was obtained from a 5 V AC adapter that was connected to a 100 V waterproof electric socket, which was originally installed in the dam. One of the signal repeaters was installed outside, and it provided wireless communication between the dam and the storage building. It was equipped with a portable solar panel to charge a built-in battery (see the inset of Figure 2c).

The developed data acquisition system enabled a sampling rate of up to 20 Hz. However, in this study, the static strain analysis was of particular interest. Thus, the sampling rate was set to 1 Hz, and the duration of each measurement was set to 10 s. After each measurement, the average of 10 measurement points was calculated, and measurements were repeated every one hour. The transmitters and signal repeaters had an integrated power management module through which their operation could be put into sleep mode. This was done to reduce the power consumption, especially that of the signal repeater installed outside, which used a battery as a power source. Sleep mode was automatically activated after each measurement.

## 3. Results

### 3.1. Long-Term Stability Test

**Figure 3:**
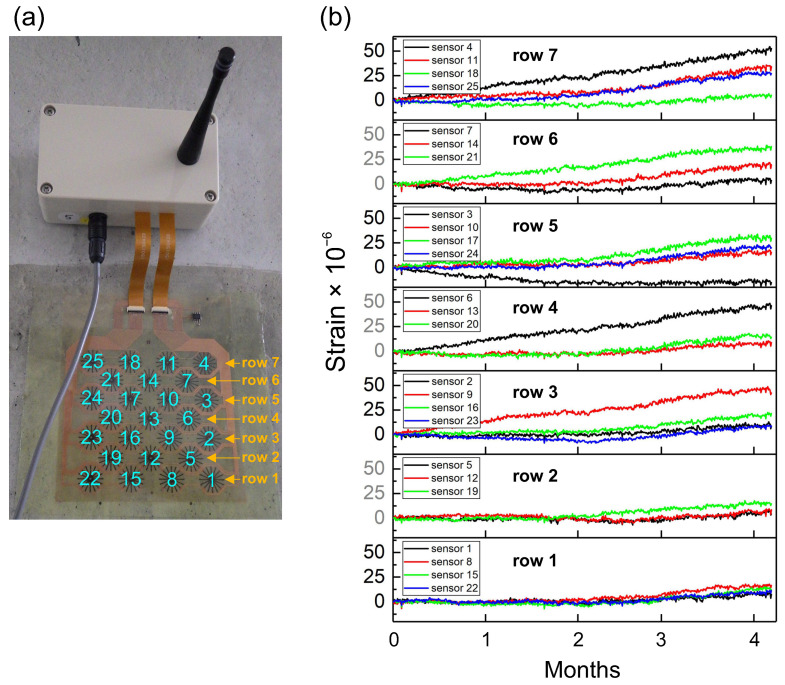
(**a**) Sensor array with the connected wireless transmitter installed on a surface with no crack. The numbers correspond to specific sensors in the array. To make it easier to distinguish data between all 25 sensors, the results were analyzed with respect to the rows in the array. (**b**) Four-month-long static strain measurement to assess the long-term stability of the sensors.

The sensor array with the connected wireless transmitter was attached to the concrete wall of the gallery using an epoxy-based adhesive sheet (Figure 3a). The developed software was run on a computer located outside the dam, and it was set to trigger the measurements automatically. During each measurement, 10 measuring points were obtained every 1 s, and the average strain value was calculated. This analysis was conducted simultaneously for all 25 sensors incorporated in the array. Figure 3b shows the results of the entire period of the four-month-long analysis. To make it easier to distinguish data between all 25 sensors, the results were split and arranged with respect to the rows in the array. The sensor array in Figure 3 was installed upside down; thus, the numbering of the rows is shown in descending order. However, the orientation of the installation did not matter in this case. Blue numbers represent the positions occupied by individual sensors in the array.

The analysis of the results showed that all 25 sensors exhibited relatively low drift, even after four months of measurement. However, it can be observed that some of the sensors were more stable than others. For example, in row 7, for sensor 18, the calculated change in the average strain over four months was only −1.9 μϵ ± 3.2 μϵ. However, in the same row, sensor 4 exhibited a slowly increasing trend for the entire measurement period. The maximum value reached after four months was 54 μϵ, and the change in the calculated average strain was 25.3 μϵ ± 14.6 μϵ.

However, the comparison of sensors in row 1 demonstrates a very uniform output signal; the measured values are almost superposed. The change in the calculated average strain for row 1 is 2.5 μϵ ± 4.6 μϵ. Among all 25 sensors in the array, the signal drift by sensor 4 was the highest; however, it can be considered as relatively low overall. For comparison, in other studies on custom-made constantan-based strain gauges, a drift of 3 μϵ over a two-day experiment was reported (1.5 μϵ per day) [14]. In this study, the sensors were analyzed over 127 days. Sensor 4 exhibited the highest drift; the recorded value was 54 μϵ (0.4 μϵ per day), which is similar to the values that were previously reported in other studies. However, it is noted that the drift was not always constant among the sensors in the array, and its trend may change. The trend of the drift and its uniformity within the sensor array may be affected by several factors, such as the quality of the fabrication process, the uniformity of the thickness of the adhesive, and the uniformity of the surface on which the sensors are installed.

**Figure 4:**
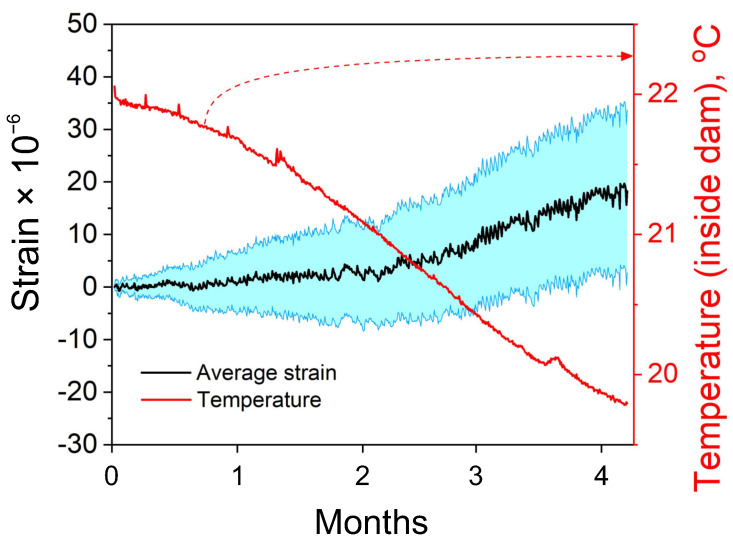
Average static strain calculated from all sensors in the array. The blue band represents the calculated error. The collected strain data are presented with the temperature measurements recorded inside the dam.

Figure 4 shows the average static strain calculated from the entire array of 25 printed strain sensors. The data are presented with the recorded temperature change inside the dam. The temperature was recorded using a temperature sensor chip that was installed on the same substrate as the printed sensors. Thus, the measured values show the local temperature changes to which the strain sensors were actually subjected. The measured maximum average strain change was only 20 μϵ. The results show that over 127 days, the temperature changed by only 2 °C, and it had a low impact on the recorded strain values. Overall, the collected data exhibits a relatively low drift over the long-term analysis. However, to assess the suitability of the developed sensors for damage detection, the results were compared with data registered on the crack in the next experiment.

### 3.2. Measurements on the Crack

**Figure 5:**
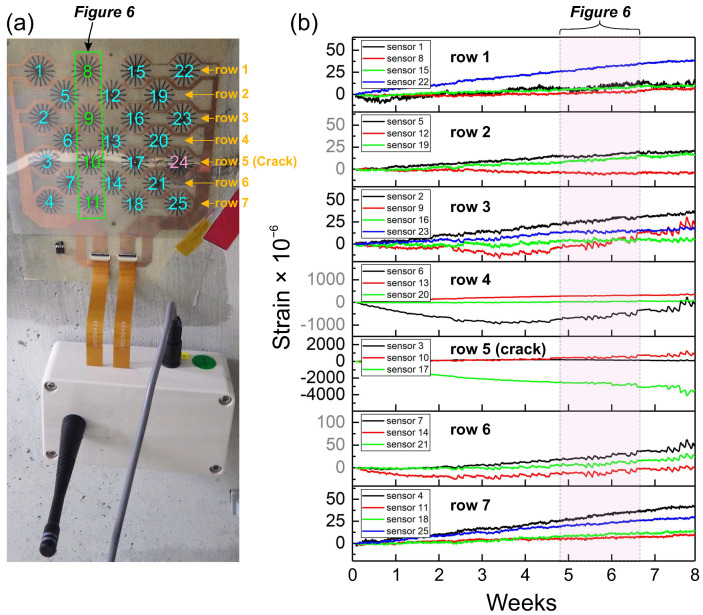
(**a**) Sensor array installed on the crack. The brightness and contrast of the photographs were changed locally to show the position of the crack. (**b**) Results of a two-month-long static strain analysis using a sensor array installed on the crack.

In the previous experiment, which was recorded on the undamaged surface, the change in the average strain that was calculated from all 25 sensors was 20 μϵ over four months (Figure 4). This shows that the developed strain sensors can generate relatively stable output signals over a long period of time. However, to assess the sensors’ capability to detect damage, based on a static strain analysis, the second sensor array was bonded to an already existing small crack on the wall of the gallery inside the dam (Figure 5a). The experiment aimed to analyze the magnitude of strain variations on the crack and to compare the results with the recorded strain changes on the undamaged surface. It was expected that the sensors installed on the crack would be subjected to larger strains caused by periodic crack opening related to water level changes in the dam. The results of the analysis are shown in Figure 5b. Compared with the previous experiment, the duration of the analysis on the crack was shorter (two months). However, even a few days of analysis is sufficient to assess the strain levels on a crack. Similar to the previous case, the recorded data were grouped with respect to the sensor rows. Sensor 24 was broken during installation; thus, it was omitted in this study. Note that underneath sensor 24, the surface was very irregular owing to the local loss of material on the wall. In such a scenario, particular attention should be paid to avoid damaging the thin-film sensor during installation.

The comparative analysis of the data recorded by the sensor array clearly exhibited the impact of the crack on the measured strain values (Figure 5b). The sensors located far from the crack (rows 1, 2, and 7) recorded similar low strain levels to those demonstrated in the previous experiment (Figure 3). It can also be observed that sensors installed a few millimeters away from the crack recorded higher strains than those located further from the crack (especially sensor 6 in a row 4). This demonstrates that indirect damage detection is possible; however, it is limited to a small distance from the damaged area. In the present construction of the sensor array, the indirect detection of cracks was very likely caused by the local transfer of stress in the adhesive and the substrate (all sensors are printed on a common substrate), which was caused by the strain changes near the crack opening.

**Figure 6:**
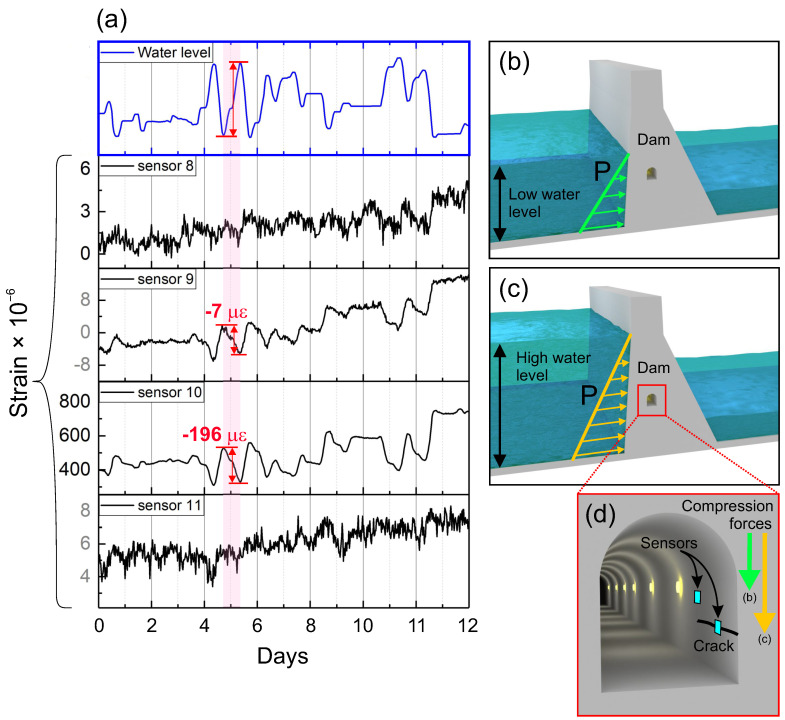
(**a**) Comparison of the measured static strain variations with recorded water level changes that were provided by the dam operator. For comparison, data from sensors 8, 9, 10, and 11 (in the same column) were selected to show the magnitude of strain variations on the crack and away from it. The water stored on the dam always exerts some pressure on the dam construction, regardless of (**b**) low or (**c**) high water level. (**d**) Illustration demonstrating the location of sensors installed on the wall of the gallery. The pressure acting on the dam causes compression forces on the crack, the magnitude of which depended on the level of water in the dam.

Among the analyzed sensors, those in row 5 (installed directly on the crack) were exposed to the greatest strains. The results demonstrate that the developed sensing system is fully capable of damage detection. Based on the long-term static strain analysis, cracks were accurately detected and localized. However, it was observed that the strain magnitude between the sensors in row 5 was different. Such differences in strain sensing may occur depending on the orientation of the installed sensors with respect to the crack direction. Strain sensors generally have one sensing direction along which they exhibit maximum sensitivity. In addition, strain sensors are typically calibrated when the entire sensor structure is subjected to uniaxial deformation. However, in the case of a crack, only a small fraction of the sensor covering a crack is exposed to large strains. Thus, sensor measurements depend on the crack orientation with respect to the direction of the sensors in the array and on the extent to which the sensing area of a sensor covers the crack. Therefore, obtaining reliable field measurements for the actual value of a crack opening remains challenging. However, an assessment of whether (and when) a crack opens or closes is possible, as shown in Figure 6.

In the recorded data, as shown in Figure 5, short-term strain variations were observed in addition to the long-term static strain evolution. Such short-term strain variations were analyzed based on four sensors selected from the array that were located in the same column (sensors 8, 9, 10, and 11). A closer view of the recorded data is shown in Figure 6a. Additionally, the measured strains were compared with the profile of water level changes in the dam. Note that the exhibited data do not provide the exact dates and values of the water level; such information is sensitive from the point of view of the dam operator and cannot be disclosed. However, this information is not relevant to the present study. Here, the profile of the changes in water level was compared with the recorded strain changes.

As expected, the highest strain change (−196 μϵ) was measured for the crack (sensor 10). However, as the water level increased, the measured strain values decreased. This was observed during the entire measurement period. The measured strains were similar to the profile of the water level changes, but with opposite signs. This observation can be associated with the specific location of the crack, as schematically illustrated in Figure 6b–d. Regardless of the level of water stored in the dam, the water always exerts some pressure on the dam construction. The recorded strain data suggest that the water pressure acting on the dam caused compression forces on the crack, the magnitude of which depended on the level of water in the dam. Similar results were observed for sensor 9, which was installed approximately 1.5 cm away from the crack. Although the measured strain variations were significantly smaller (only −7 μϵ), the profile of the strain changes was also similar to the water level changes. However, sensors 8 and 11, which were installed further or on the opposite side of the crack, exhibited almost unnoticeable daily strain variations. These results are similar to those recorded by the sensor array that was installed on the undamaged surface. This indicates that the 2D strain sensor arrays are capable of damage detection, both directly on the crack and indirectly, but only if located near a crack. However, considering their relatively large sensing area compared with that of conventional strain sensors, 2D sensor arrays may be especially suitable for monitoring sensitive construction elements of the analyzed engineering structures. The layout of the sensors and their sizes can be easily scaled up in future research using printing methods. Overall, the above-mentioned results show that the printed strain sensors are sensitive enough for damage detection, and they exhibit relatively low signal drift. Therefore, they are suitable for long-term static strain measurements in monitored civil engineering structures.

## 4. Conclusions

This study evaluated printed strain sensors based on long-term static strain measurements conducted inside a gravity dam. The analysis assessed the long-term stability of printed sensors and their suitability for practical implementation in static strain analysis within the framework of SHM.

The recorded data exhibited an average signal drift of 20 μϵ over 127 days (less than 1 μϵ per day). Such a drift level is considered low, especially when compared with the results registered on the existing crack. The measurements on the crack showed that the changes in daily strain could be as high as 196 μϵ. However, lower daily strain variations, owing to the crack opening driven by water level changes, could be detected by the sensors that were located near the crack. Although indirect damage detection is possible, it is limited to a small distance from the damaged area. Nonetheless, determining the exact values of such a detection limit is very difficult because it depends on the crack opening and orientation of the installed sensors with respect to the crack direction. For this reason, large sensors arranged into dense sensor arrays are more suitable for damage detection.

Overall, the printed sensors exhibited good strain sensitivity and relatively low signal drift. The results show that printed sensors incorporated into automated DAQ can be used as complementary sensing systems to those already existing within the framework of SHM.
